# Genomic Island-Encoded Diguanylate Cyclase from *Vibrio alginolyticus* Regulates Biofilm Formation and Motility in *Pseudoalteromonas*

**DOI:** 10.3390/microorganisms11112725

**Published:** 2023-11-08

**Authors:** Tongxuan Cai, Huan Tang, Xiaofei Du, Weiquan Wang, Kaihao Tang, Xiaoxue Wang, Dong Liu, Pengxia Wang

**Affiliations:** 1 Key Laboratory of Tropical Marine Bio-Resources and Ecology, Guangdong Key Laboratory of Marine Materia Medica, Innovation Academy of South China Sea Ecology and Environmental Engineering, South China Sea Institute of Oceanology, Chinese Academy of Sciences, No.1119, Haibin Road, Nansha District, Guangzhou 511458, China; ctxyydsyyds@163.com (T.C.); th429788@163.com (H.T.); xh13233909964@163.com (X.D.); wangweiquan@scsio.ac.cn (W.W.); khtang@scsio.ac.cn (K.T.); xxwang@scsio.ac.cn (X.W.); 2 College of Life Sciences, Hebei Normal University, Shijiazhuang 050024, China; 3 Southern Marine Science and Engineering Guangdong Laboratory (Guangzhou), No.1119, Haibin Road, Nansha District, Guangzhou 511458, China; 4 University of Chinese Academy of Sciences, Beijing 100049, China

**Keywords:** mobile genetic elements, *Vibrio*, *Pseudoalteromonas*, motility, biofilm

## Abstract

Many bacteria use the second messenger c-di-GMP to regulate exopolysaccharide production, biofilm formation, motility, virulence, and other phenotypes. The c-di-GMP level is controlled by the complex network of diguanylate cyclases (DGCs) and phosphodiesterases (PDEs) that synthesize and degrade c-di-GMP. In addition to chromosomally encoded DGCs, increasing numbers of DGCs were found to be located on mobile genetic elements. Whether these mobile genetic element-encoded DGCs can modulate the physiological phenotypes in recipient bacteria after horizontal gene transfer should be investigated. In our previous study, a genomic island encoding three DGC proteins (Dgc137, Dgc139, and Dgc140) was characterized in *Vibrio alginolyticus* isolated from the gastric cavity of the coral *Galaxea fascicularis*. Here, the effect of the three DGCs in four *Pseudoalteromonas* strains isolated from coral *Galaxea fascicularis* and other marine environments was explored. The results showed that when *dgc137* is present rather than the three DGC genes, it obviously modulates biofilm formation and bacterial motility in these *Pseudoalteromonas* strains. Our findings implied that mobile genetic element-encoded DGC could regulate the physiological status of neighboring bacteria in a microbial community by modulating the c-di-GMP level after horizontal gene transfer.

## 1. Introduction

Horizontal gene transfer is an important driving factor for the adaptation and evolution of bacterial communities, because it accelerates the exchange of genetic material between different strains or species [1]. Mobile genetic elements, such as prophages, plasmids, integrative and conjugative elements (ICEs), and genomic islands, are the main vectors that mediate horizontal gene transfer by delivering adaptive genes [2,3]. Horizontal gene transfer is thought to be pervasive in the coral microbiome, and offers significant and greatly unrecognized potential for the adaptive capacity of the coral microbiome to a rapidly changing environment [4].

*Vibrio*, *Pseudoalteromonas*, *Alteromonas*, and *Shewanella* are widely distributed in various marine environments, and these species often contain similar mobile genetic elements in their genomes [5,6,7,8]. These genera cohabit in the same ecological niches, e.g., the gastric cavity of coral [8], seawater, marine sediments, and hydrothermal vents [9], which provides the prerequisites for horizontal gene transfer among these species. ICEs of the SXT/R391 family are a large class of mobile genetic elements that play important roles in horizontal gene transfer among these species. SXT/R391 ICEs have been defined based on site-specific integration into *prfC*, a gene encoding peptide chain release factor 3. In recent years, atypical SXT/R391 ICEs have also been found to be integrated at a serine tRNA gene in *Vibrio alginolyticus* [10] or integrated at the *pabB* gene encoding para-aminobenzoate synthase component II in *Pseudoalteromonas* [8]. SXT/R391 ICEs are not only self-transmissible elements like conjugative plasmids, but can also integrate into and excise from the host chromosome [11]. Moreover, they can also drive the mobilization of a class of genomic islands integrated in the *yicC* gene [12], or transfer a tandem genomic island in *cis* among these species [7]. In addition to SXT/R391 ICEs, prophages or genomic islands integrated into the tRNA modification enzyme-encoding gene *trmE*, tRNA (uridine(54)-C5)-methyltransferase-encoding gene *trmA*, *ssrA* (small stable RNA A), or other tRNA-related genes were also characterized in these species [8,13,14]. The most attention to the cargo genes in these mobile genetic elements has been paid to the dissemination of antimicrobial resistance genes and heavy metal resistance across bacterial genomes [15,16]. The diversity of the functions of cargo genes remains seriously neglected.

Diguanylate cyclases (DGCs) are responsible for the synthesis of the second messenger cyclic di-guanosine monophosphate (c-di-GMP), which is a global bacterial second messenger that can regulate diverse physiological phenotypes [17]. DGCs have a conserved catalytic GGDEF domain that plays a key role in their activity. With an opposite function, bacteria also encode phosphodiesterases (PDEs), which are responsible for the degradation of c-di-GMP via an EAD or HD-GYP domain [18]. More than 60 chromosomally encoded genes in *Vibrio cholerae* related to c-di-GMP [19] and 45 putative GGDEF domain-containing proteins were found in *Vibrio alginolyticus* SCSIO 43097 (abbreviated Va43097) [8]. The redundancy of DGCs and PDEs in controlling cellular processes indicated the complexity of c-di-GMP regulation.

In addition to chromosomally encoded DGC genes, some DGCs were also found to be located on mobile genetic elements as cargo genes. Genes *dgcK* and *dgcL* were characterized in ICE*Vch*Mex1 belonging to SXT/R391 ICEs in *V. cholerae* 1-010118-075 and are frequently found in other SXT/R391 ICEs, including ICE*Vfl*Ind1 in *Vibrio fluvialis* H-08942 and ICE*Vch*Moz3 in *V. cholerae* 7AMOZ. Overexpression of DgcK or DgcL could modulate gene expression, biofilm formation, and bacterial motility [19]. Our previous study showed tight cooperation of SXT/R391 ICEs and mobilizable genomic islands integrated in *yicC* from *Pseudoalteromonas* spp. mediated the excision of the genomic island VPII integrated in *trmA* of *Vibrio alginolyticus* after horizontal gene transfer. The loss of VPII significantly reduced biofilm formation and phage resistance, but increased bacterial motility. VPII encoded three DGCs: Dgc137, Dgc139, and Dgc140, and Dgc137 was proven to be the main factor responsible for these phenotypic changes in its original host bacteria. Genes *dgc137*, *dgc139,* and *dgc140*-bearing genomic islands were also distributed in *Vibrio diabolicus* FDAARGOS_96 *and V. alginolyticus* ANC4-19 [8]. These reports demonstrated that the DGCs carried by mobile genetic elements can modulate the physiological phenotypes of their original host, similar to chromosomally encoded DGCs. As cargo genes, these DGC genes can be spread as antibiotic resistance genes by horizontal gene transfer. It is still unclear whether these DGCs can modulate the physiological phenotypes of recipient bacteria after horizontal gene transfer.

Here, to explore the effect of mobile genetic element-encoded DGCs in recipient strains after horizontal gene transfer, VPII-encoded DGCs were expressed in four *Pseudoalteromonas* species, which always cohabit in the same ecological niches as *Vibrio*. Two plasmids expressing a single Dgc137 or expressing three DGCs (Dgc137, Dgc139, and Dgc140) under their native promoters were constructed and transferred into four different *Pseudoalteromonas* strains. The results showed that the expression of Dgc137 modulates biofilm formation and bacterial motility in these *Pseudoalteromonas* strains. Our findings reveal that DGC proteins delivered by genomic islands could control the physiological phenotype of host bacteria after horizontal gene transfer.

## 2. Materials and Methods

### 2.1. Bacterial Strains

The bacterial strains and plasmids used in this study are listed in Table 1. *Vibrio* and *Pseudoalteromonas* strains were grown in 2216E medium (Difco) or SWLB (10 g peptone, 5 g yeast extract, and 1000 mL seawater) at 30 °C. *Escherichia coli* WM3064 was grown in LB medium containing 0.3 mM DAP (2,6-diamino-pimelic acid) at 37 °C. Modified LB medium (10 g peptone, 5 g yeast extract, 500 mL seawater, and 500 mL distilled water) with 0.3 mM DAP was used in conjugation assays. Cm (chloramphenicol, 30 μg mL^−1^) was used in *E. coli*, while 15 μg mL^−1^ Cm was used in *Pseudoalteromonas*.

### 2.2. Plasmid Construction

In plasmid pDgc137, a 1.4-kb fragment that contained the *dgc137* gene and its promoter was amplified from Va43097 and inserted into the *Eco*RI/*Kpn*I sites of plasmid pBBR1Cm [8]. Similarly, a 5.3-kb fragment containing *dgc137, dgc139,* and *dgc140* was amplified from Va43097 using primers p3DGC-F (GTGACCGTGTGCTTCGAATTCGTGTTACTCTTTATCTGAGCG) and p3DGC-R (GGGAACAAAAGCTGGGTACCCTTGAAATCAGGTATTAGGAAG) and inserted into the same sites of plasmid pBBR1Cm using the ClonExpress II One Step Cloning Kit (Vazyme Biotech Co., Ltd.). The correct constructs were confirmed by PCR amplification and sequencing.

### 2.3. Conjugation Assays

The plasmids pDgc137 and p3DGC and the empty vector pBBR1Cm were transferred to *Pseudoalteromonas* using conjugation assays, as described previously [23]. Briefly, plasmids pDgc137, p3DGC, and pBBR1Cm were transformed into *E. coli* WM3064 to generate the donor strain, and *Pseudoalteromonas* was used as the recipient strain. Then, equal amounts of donor and recipient strains were harvested and dropped on modified LB agar medium after mixing. The plates were incubated for 8–12 h until a bacterial lawn was formed at 30 °C. Cells were collected from the lawn and streaked on 2216E plates with Cm to select the transconjugants.

### 2.4. Observation of Colony Morphology

The tested bacteria were inoculated overnight, and then 10 μL of overnight culture was spotted onto 2216E agar plates and incubated for 3–6 days at 30 °C. Colony morphology was imaged using stereoscopic microscopy.

### 2.5. Motility and Biofilm Assays

Swimming motility was measured using semisolid agar plates containing 0.3% agar (*w*/*v*) in 2216E medium after culturing for 15–52 h at 25 °C. Crystal violet staining was used to evaluate the amount of attached biofilm in 96-well polystyrene plates by measuring OD_540_ and OD_620_ [24]. The tested bacteria were stationary cultured in test tubes containing 2216E medium to form pellicles. Pellicle morphology was observed on the air–liquid interface of the medium and photographed at 36 h.

### 2.6. Prediction of GGDEF Domain-Containing Proteins

PFAM domain annotation of *Pseudoalteromonas* strains was performed using eggNOG-mapper v2 [25] (http://eggnog-mapper.embl.de, accessed on 27 September 2023) by uploading annotated genomes. GGDEF domain-containing proteins can be obtained from the generated files.

### 2.7. Statistics and Reproducibility

Data analyses were performed using GraphPad Prism 9 XML Project. The quantitative data were analyzed using unpaired t tests for two-sample comparisons. Asterisks represent statistically significant differences (ns, (not significant); * *p* < 0.05; ** *p* < 0.01; *** *p* < 0.001; **** *p* < 0.0001). Individual data points are plotted with lines at the mean, and error bars represent the standard deviation in each figure. Biological replicates are shown in the figure legends.

## 3. Results

### 3.1. Dgc137 Regulates the Colony Biofilm of Pseudoalteromonas

**Figure 1:**
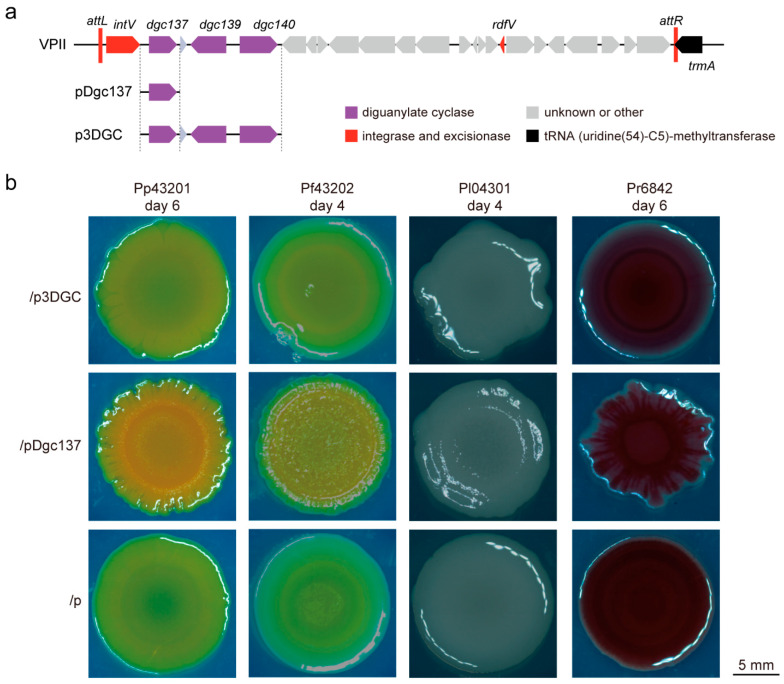
The change in colony biofilm formation when DGC137 and three DGC proteins were expressed in *Pseudoalteromonas* strians. (**a**) Construction of pDgc137 and p3DGC. The DNA regions contained in pDgc137 and p3DGC are indicated as solid lines with the ORFs, and the end of the DNA region is indicated as dashed lines. (**b**) The change in colony biofilm in the indicated *Pseudoalteromonas* strains containing pDgc137 and p3DGC compared with the empty vector. “p” indicates the empty vector pBBR1Cm. At least three independent cultures were used, and only representative images are shown. The incubation days of the colony biofilm imaged are indicated under the strain name.

Three DGC enzymes, Dgc137, Dgc139, and Dgc140 are encoded by VPII, and Dgc137 was proven to be the main factor regulating swimming and biofilm formation in Va43097 [8]. Thus, two plasmids, pDgc137 and p3DGC, were constructed. In plasmid pDgc137, a fragment containing the *dgc137* gene with its native promoter was cloned in pBBR1Cm [8], and in p3DGC, a fragment containing all three DGC genes *dgc137*, *dgc139,* and *dgc140* with their native promoters was cloned in the same sites of pBBR1Cm (Figure 1a). Then, four *Pseudoalteromonas* strains, including *Pseudoalteromonas peptidolytica* SCSIO 43201 (abbreviated Pp43201), *Pseudoalteromonas flavipulchra* SCSIO 43202 (abbreviated Pf43202), *Pseudoalteromonas lipolytica* SCSIO 04301 (abbreviated Pl04301), and *Pseudoalteromonas rubra* SCSIO 6842 (abbreviated Pr6842), representing different species, were selected as the recipient strains for DGC expression. These four strains were isolated from different ecological habitats in the neritic region; for example, Pp43201 and Pf43202 were isolated from the gastric cavity of the coral *Galaxea fascicularis* on Hainan Island, which is the same coral species as Va43097. Pl04301 was isolated from sediment at a depth of 63 m in the South China Sea, and Pr6842 was isolated from 150 m below the surface in the Bay of Bengal (Table 1). All four *Pseudoalteromonas* strains contain two chromosomes, and Pp43201 and Pr6842 also contain a plasmid. Pp43201, Pf43202, and Pr6842 are pigmented, and Pl04301 is nonpigmented. By searching the conserved domain at the eggNOG-mapper web, more than 50 putative GGDEF domain-containing proteins were found in the four *Pseudoalteromonas* strains. Among them, the Pl04301 genome encodes the most, with 68 GGDEF domain-containing proteins (Table 2 and Supplementary Materials Datasets 1–4).

Conjugation assays were performed to transfer the plasmids pDgc137 and p3DGC into the *Pseudoalteromonas* strains, and the empty vector pBBR1Cm was used as a control, generating four groups of *Pseudoalteromonas* strains bearing pDgc137, p3DGC, and pBBR1Cm. Then, these *Pseudoalteromonas* strains were grown on 2216E agar plates at 25 °C, and the formation of colony biofilms was observed. After culturing the strains for 4–6 days, the colonies were smooth in all pBBR1Cm-bearing *Pseudoalteromonas* strains, but the colonies were wrinkled in Pp43201, Pf43202, and Pl04301 and were striped in Pr6842 when pDgc137 was present. However, there were no significant changes in colony morphology when p3DGC was present in these *Pseudoalteromonas* compared with the empty vector (Figure 1b). These results suggested that the expression of Dgc137 alone can regulate the colony biofilms of *P. peptidolytica*, *P. flavipulchra*, *P. lipolytica,* and *P. rubra*, but simultaneous expression of the three DGC proteins cannot.

### 3.2. Dgc137 Increases the Attached Biofilm of Pseudoalteromonas

**Figure 2:**
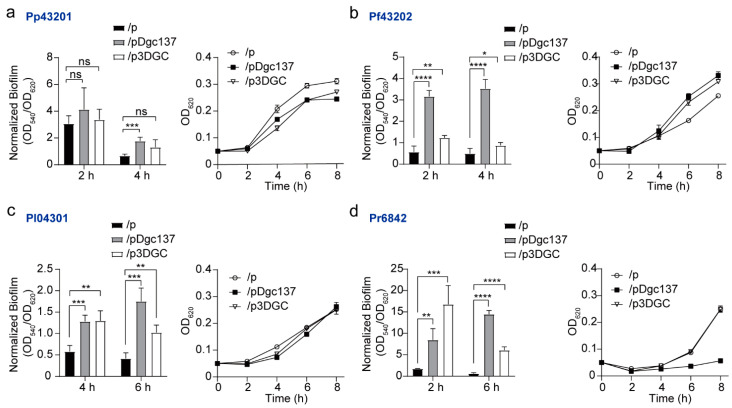
The change in biofilm formation and growth when the indicated *Pseudoalteromonas* strains contained pDgc137 and p3DGC compared with the empty vector pBBR1Cm. Biofilm formation and growth of Pp43201 (**a**), Pf43202 (**b**), Pl04301 (**c**), and Pr6842 (**d**) when pDgc137, p3DGC, and pBBR1Cm were expressed. “p” indicates pBBR1Cm. The host bacteria are indicated at the top of each panel. All the tested bacteria were cultured in 2216E medium for 0–8 h, and only the data from representative time points are shown. Individual data points are plotted with lines at the mean, and error bars represent the standard deviation. Statistical analysis: Unpaired t-test, asterisks represent statistically significant differences (ns, (not significant); * *p* < 0.05; ** *p* < 0.01; *** *p* < 0.001; **** *p* < 0.0001).

In addition to colony biofilms, *Pseudoalteromonas* strains commonly form attached biofilms at liquid‒solid interfaces and pellicles at air‒liquid interfaces. The changes in the attached biofilms of these *Pseudoalteromonas* strains were then analyzed using a 96-well microplate assay. As shown in Figure 2, expressing pDgc137 increased biofilm formation of all four *Pseudoalteromonas* strains to different degrees. When pDgc137 was present, biofilm formation increased by 2.7 ± 0.4-fold at 4 h in Pp43201, 6.4 ± 1.8-fold at 4 h in Pf43202, 4.4 ± 0.8-fold at 6 h in Pl04301, and 25.4 ± 7.8-fold at 6 h in Pr6842. When p3DGC was present, biofilm formation increased by 2.3 ± 1.7-fold at 4 h in Pf43202, 2.6 ± 0.7-fold at 6 h in Pl04301, and 10.4 ± 2.3-fold at 6 h in Pr6842, but had no significant effect on biofilm formation in Pp43201 at 2 h and 4 h.

By tracking the OD_620_ of these *Pseudoalteromonas* strains cultured in 96-well microplates at 0–8 h, we found that expressing pDgc137 and p3GDC had no significant effect on the growth of Pl04301. Notably, containing pDgc137 and p3GDC decreased the growth of Pp43201 but increased the growth of SCSIO 43202. Meanwhile, pDgc137 sharply decreased the growth of Pr6842, but p3DGC did not affect the growth of Pr6842.

### 3.3. Dgc137 Increases the Pellicle of Pseudoalteromonas

**Figure 3:**
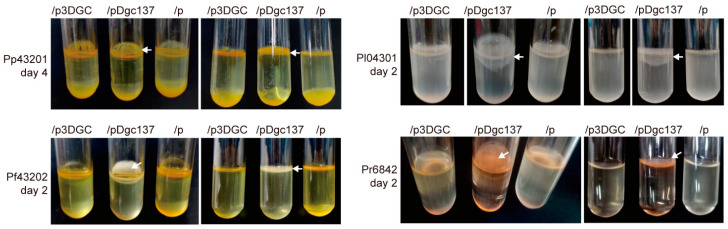
The change in pellicle formation when the indicated *Pseudoalteromonas* strains containing pDgc137 and p3DGC were compared with the empty vector. “p” indicates the empty vector pBBR1Cm. The white arrows indicate floating pellicles. The incubation days of the pellicle imaged are also shown on the left of the image. At least three independent cultures were used, and only representative images are shown.

To assess the changes in pellicle formation, the strains were cultured statically in test tubes with 2216E liquid medium for 2–4 days. The four *Pseudoalteromonas* strains formed more floating pellicles when containing pDgc137 than when containing the empty vector pBBR1Cm. However, containing p3DGC did not show an obvious effect on the pellicle formation of the four *Pseudoalteromonas* strains (Figure 3). Collectively, DGC137 increases biofilm and pellicle formation in *P. peptidolytica*, *P. flavipulchra*, *P. lipolytica,* and *P. rubra* to different degrees.

### 3.4. Dgc137 Decreases the Motility of Pseudoalteromonas

**Figure 4:**
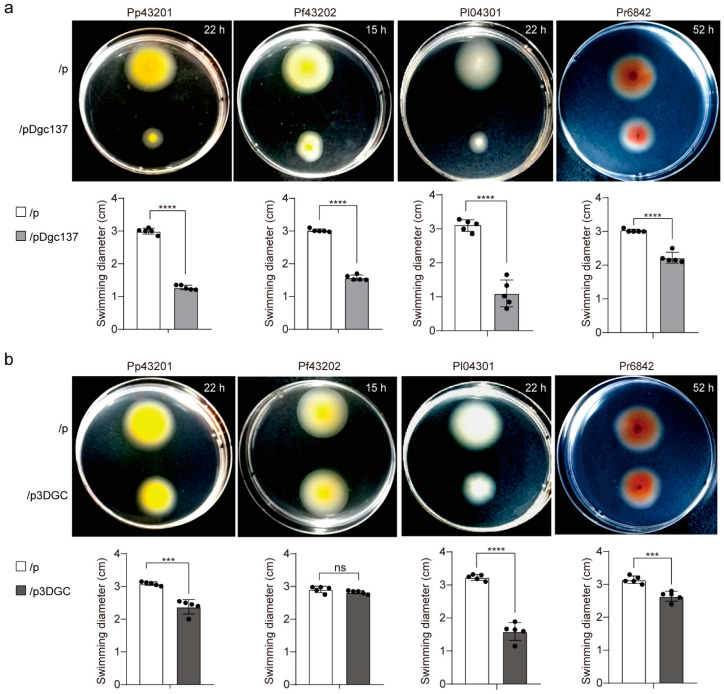
The change in swimming ability when the indicated *Pseudoalteromonas* strians contained pDgc137 and p3DGC compared with the empty vector. Swimming diameter when the indicated *Pseudoalteromonas* strains containing pDgc137 (**a**), and p3DGC (**b**) were compared with the empty vector. “p” indicates the empty vector pBBR1Cm. The plates were imaged after culturing the strains in semisolid agar plates for 22 h (Pp43201), 43202 15 h (Pf43020), 22 h (Pl04301), and 52 h (Pr6842). At least three independent cultures were used, and only representative images are shown. Quantitative statistical data are shown under the representative images. Statistical analysis: Unpaired *t*-test, asterisks represent statistically significant differences (ns, (not significant); - *** *p* < 0.001; **** *p* < 0.0001).

Our previous study showed that Dgc137 and Dgc139 regulate swimming ability in Va43097 [8]. Therefore, a motility assay was also performed using semisolid agar plates. As shown in Figure 4, the four *Pseudoalteromonas* strains exhibited different motility abilities, and SCSIO 6842 exhibited the slowest swimming ability on semisolid agar. Therefore, these strains were incubated for different incubation times before imaging. Expressing pDgc137 significantly decreased the swimming ability of all four *Pseudoalteromonas* strains. Pp43201 containing pDgc137 (halo diameter of 1.3 ± 0.1 cm) had a significantly smaller swimming ability than that without the *dgc137* gene (3.0 ± 0.1 cm), decreasing 2.4 ± 0.1-fold. Similarly, the halo diameter decreased by 1.9 ± 0.1-fold in Pf43202, 3.1 ± 1.1-fold in Pl04301, and 1.4 ± 0.1-fold in Pr6842 when pDgc137 was present compared with the control.

In contrast, the presence of p3DGC results in a different effect on the swimming ability of the four *Pseudoalteromonas* strains. With p3DGC, the halo diameter had the greatest change (3.2 ± 0.1 cm) in Pl04301, which decreased by 2.1 ± 0.4-fold compared to the empty vector (1.6 ± 0.3 cm), and decreased by 1.2 ± 0.1-fold in Pp43201 and Pr6842 when p3DGC was present. No significant difference was observed in the swimming ability of Pf43202 when p3DGC was present compared with pBBR1Cm (Figure 4b). These results suggested that DGC137 decreased swimming motility in the strains *P. peptidolytica*, *P. flavipulchra*, *P. lipolytica,* and *P. rubra*, whereas when the plasmid containing the three DGCs was conjugated, the effects depended on the strain.

## 4. Discussion

*Pseudoalteromonas* and *Vibrio* are widely distributed genera in coral and other marine environments [8,9,29], and commonly form biofilms to resist environmental stresses. Switching between biofilm and motile lifestyles is an important decision for them to adapt to changing environments and could be regulated by the c-di-GMP level [30,31]. This study provides evidence to show that a genomic island-encoded DGC protein from a coral-associated *Vibrio alginolyticus* could promote the transition of the motile style to the sessile style of *Pseudoalteromonas* strains. We propose that horizontal gene transfer might not only contribute to the dissemination of antimicrobial resistance genes and virulence genes as cargos, but also arbitrarily determine the physiological status of neighboring bacteria in a microbial community.

DGCs and PDEs are c-di-GMP metabolism proteins that modulate c-di-GMP levels [31]. A puzzling question in the study of c-di-GMP is why the bacterial cell possesses so many DGC and PDE proteins and how the bacteria integrate their contributions. In addition to SXT/R391 ICEs and VPII, DGC-encoding genes have been identified in other ICEs, bacteriophages, and plasmids [8,19]. This brings greater challenges in understanding the complexity of c-di-GMP regulation with the discovery of DGCs in increasing mobile genetic elements. DGC activity for the biosynthesis of c-di-GMP is catalyzed by a GGDEF domain. All the DGC proteins used in this study, Dgc137, Dgc139, and Dgc140, bear a GGDEF domain. Similar to its original *Vibrio* host, expressing Dgc137 had a more obvious effect than expressing the three DGCs on biofilm formation and motility in *Pseudoalteromonas*. Notably, the swimming ability decreased in all four *Pseudoalteromonas* strains, and the formation of colony biofilms, attached biofilms, and pellicles increased when Dgc137 was expressed. Dgc137 also affected the growth of Pp43201, Pf43202, and Pr6842 but had no influence on the growth of Pl04301, indicating that Dgc137 is involved in the varied physiological metabolism of *Pseudoalteromonas*. For Pr6842, expressing Dgc137 also affected the production of pigment. All these results indicated that the function of a DGC protein varies in different bacteria. This phenomenon is also found in the VieA protein, which enhances motility and represses biofilm formation in the classical biotype, but not in E1 Tor [32].

In addition to containing a GGDEF domain, many DGC proteins contain putative EAL domains or sensory domains, such as the Per/Arnt/Sim (PAS), GAF, CHASE, REC, and HK_sensor domains, adjacent to their GGDEF domain [18,33,34]. Dgc137 contains a PAS domain coupled with a GGDEF domain. Dgc139 contains both a GGDEF domain and an EAL domain, and Dgc140 contains a GGDEF domain coupled with two HK_sensor domains at the amino terminus [8]. In this study, more than 50 putative GGDEF domain-containing proteins were predicted in Pp43201, Pf43202, Pl04301, and Pr6842. How these proteins regulate biofilm formation and motility is unclear. To fully understand the function of Dgc137, Dgc139, and Dgc140 in these *Pseudoalteromonas*, their contributions to cellular c-di-GMP metabolism, the integrated pathways, and the environmental signals they sense should be further determined.

In our previous study, deletion of *dgc137* resulted in similar phenotypic changes in biofilm formation and bacterial motility as deletion of VPII harboring *dgc137*, *dgc139,* and *dgc140*, suggesting that dgc137 plays a critical role in the regulation of biofilm formation and motility in *V. alginolyticus*. Transcriptome sequencing showed that the expression of *dgc137*, *dgc139,* and *dgc140* is differentially expressed in *V. alginolyticus*, and the mRNA level of *dgc137* is 3–5-fold higher than that of *dgc139* and *dgc140* [8]. In this study, simultaneous expression of Dgc137, Dgc139, and Dgc140 did not affect pellicle formation in any of the four *Pseudoalteromonas* strains but affected the colony biofilm, attached biofilm formation, and motility in some of them. These results indicated that the introduction of Dgc137 and Dgc140 might partly cancel out the activity of Dgc137 in these *Pseudoalteromonas*. Here, *dgc137*, *dgc139,* and *dgc140* are also driven by their native promoters, whereas the expression of these genes in these *Pseudoalteromonas* should be further examined. Furthermore, bacterial activity in biofilms can organize complex cellular differentiation in space and time [35]. Manner et al. discovered that *Pseudomonas aeruginosa* drives the heterogeneity of c-di-GMP signaling in biofilms so that they can undertake division of labour strategies to control surface colonization [36,37]. Whether the three DGCs are involved in cellular differentiation during biofilm formation of *Pseudoalteromonas* should be further investigated.

## Figures and Tables

**Table 1:** Strains and plasmids used in this study.

Strains/Plasmids	Description	Reference
Va43097	*Vibrio alginolyticus* SCSIO 43097, isolated from the gastric cavity of coral *Galaxea fascicularis* in China: Hainan Island	[8]
Pp43201	*Pseudoalteromonas peptidolylytica* SCSIO 43201, isolated from the gastric cavity of coral *Galaxea fascicularis* in China: Hainan Island	[20]
Pf43202	*Pseudoalteromonas flavipulchra* SCSIO 43202, isolated from the gastric cavity of coral *Galaxea fascicularis* in China: Hainan Island	[20]
Pl04301	*Pseudoalteromonas lipolytica* SCSIO 04301, isolated from sediment at 63 m deep in the South China Sea (18°0′ = N, 109°42′ = E)	[21]
Pr6842	*Pseudoalteromona rubra* SCSIO 6842, isolated from 150 m below surface at Bay of Bengal	[22]
VPII	Genomic island integrated in *trmA* of Va43097	[8]
pBBR1Cm	pBBR1MCS-Cm, broad host range cloning vector containing chloramphenicol resistance gene	[21]
pDgc137	DNA fragment containing gene *dgc137* with its promoter cloned and inserted into pBBR1Cm	[8]
p3DGC	DNA fragment containing genes *dgc*137 to *dgc*140 of VPII cloned and inserted into pBBR1Cm	This study

**Table 2:** Genetic information of the four *Pseudoalteromonas* strains used in this study.

Strains	Genome Content	Size (Mb)	Predicted Genes	GGDEF Domain-Containing Proteins ^a^	Accession Numbers	Reference
Pp43201	2 chromosomes and 1 plasmid	5.2	4374	51	CP072683.1, CP072684.1, CP072685.1	[20,26]
Pf43202	2 chromosomes	5.4	4705	55	CP024625.1, CP024626.1	[20,26]
Pl04301	2 chromosomes	4.7	4220	68	GCA_000576675.1	[27]
Pr6842	2 chromosomes and 1 plasmid	5.9	4998	51	CP013611.1, CP013612.1, CP013613.1,	[22,28]

^a^, The GGDEF domain-containing proteins in Pp43201, Pf43202, Pl04301, and Pr6842 are listed in Supplementary Materials Datasets 1–4.

## Data Availability

The authors declare that the data supporting the findings of this study are available within the article.

## Supplementary Materials

The following supporting information can be downloaded at: https://www.mdpi.com/article/10.3390/microorganisms11112725/s1, and PFAMs of GGDEF domain-containing proteins in Pp43201, Pf43202, Pl04301, and Pr6842 were included.
